# HHEX: A Crosstalker between HCMV Infection and Proliferation of VSMCs

**DOI:** 10.3389/fcimb.2016.00169

**Published:** 2016-11-30

**Authors:** Lingfang Li, Meitong Liu, Leitao Kang, Yifan Li, Ziyu Dai, Bing Wang, Shuiping Liu, Liyu Chen, Yurong Tan, Guojun Wu

**Affiliations:** ^1^Department of Vasculocardiology, Xiangya Hospital, Central South UniversityChangsha, China; ^2^Department of Microbiology, School of Basic Medical Sciences, Central South UniversityChangsha, China

**Keywords:** human cytomegalovirus, VSMCs, HOX gene, vascular proliferative diseases, haematopoietically expressed HOX

## Abstract

**Objective:** The study was designed to evaluate the role of Human cytomegalovirus (HCMV) infection on homebox (HOX) gene expression and the effects of overexpression of HOX genes on proliferation and apoptosis of vascular smooth muscle cells (VSMCs).

**Methods:** Viral infection was verified by observation of cytopathic effects through inverted microscopy, viral particles by electron microscopy and HCMV IE gene amplification by RT-PCR. cDNA profiling technology was used to screen expression of HOX genes after HCMV infection in VSMCs. Abnormal expression of Haematopoietically-expressed homeobox (HHEX) was selected to construct over-expressed vector and transfected into VSMCs. The effects of over expression of HHEX on cell proliferation and apoptosis of VSMCs were assayed by flow cytometry. Apoptosis and proliferation-associated genes were also assayed by RT-PCR.

**Results:** Multiple HOX gene expression levels had obvious changes after HCMV infection, among which expression of HHEX gene increased obviously at 24, 48, and 72 h after infection. Over expression of HHEX can promote VSMCs proliferation by promoting G0/G1 phase cells into S phase and inhibit VSMCs apoptosis. HHEX inhibited the expression of apoptosis-associated caspase 2 and caspase3 and promoted the expression of cell cycle-related genes such as CDK2 and CDK6, CyclinB2 and CyclinD2.

**Conclusion:** HHEX over expression induced by HCMV infection closely associated with vascular proliferative diseases.

## Introduction

Transplantation is the moving of an organ from one body to another or from a donor site to another location on the person's own body, to replace the recipient's damaged or absent organ. With the continuous development of medical technology, transplantation has become an important and effective treatment after organ failure. Moreover, the development of immunosuppressive agents and its application in clinical practice has largely been overcomed the acute rejection of transplantation. As a result short-term survival rate of recipients has significantly increased. But the long-term survival in patients has no obvious improvement (Tantravahi et al., 2007). This is mainly because chronic rejection deciding the long-term survival of patients has not been effectively resolved (Sarraj et al., 2014). Histologically, chronic rejection process is characterized by atrophy, fibrosis, and arteriosclerosis. Both immune and nonimmune mechanisms are likely involved in chronic rejection. Chronic rejection is often induced by graft atherosclerosis (AS), which forms arterial neointimal structure caused by excessive proliferation of vascular smooth muscle cells (VSMCs), the infiltration of inflammatory cells and the accumulation of extracellular matrix (Rahmain et al., 2006). Existing research shows that intimal abnormal proliferation plays a very important role in the occurrence and development of AS and graft AS (Ross, 1999; Autieri, 2003).

Human cytomegalovirus (HCMV) infection has close relationship with graft AS and other AS. In middle and late of the last century, experiment was performed in Fabricant induced chicken by using bird Marek's virus whose pathological changes were similar to human atherosclerosis (Fabricant et al., 1978; Fabricant and Fabricant, 1999). After this a lot of studies were done which reports that HCMV infection is one of the important pathogenic factors of AS. Moreover integrating the scholar's reports, it is found that HCMV participates in the AS through many ways including inflammatory reaction through infection of endothelial cells and VSMCs, enhanced proliferation and migration of VSMCs, inhibiting apoptosis of vascular endothelial cells and VSMCs, abnormal expression of cytokines, cell lipid metabolic disorders and body's immune damage and so on.

Cardiac allograft vascular disease (also called graft artery diseases) is characterized by accelerated and diffuse intimal proliferation involving both the microvasculature and epicardial vessels. It is the most common cause of death for recipients and also the main cause of heart re-transplantation. There are many reasons for the onset of cardiovascular diseases, but the key factor is vascular endothelial injury of the graft induced by a variety of factors including ischemia-reperfusion, allograft immune response and pathogen infection (Valantine, 2003). Studies have shown that HCMV is an important cause of graft artery diseases (Hussain et al., 2007). In solid organ transplantation, HCMV infection can promote chronic rejection and are closely related to allograft vascular diseases (Delgado et al., 2015). Animal experiments also show that the HCMV can promote the occurrence of graft vascular diseases (Suzuki et al., 2002). Pathological studies showed that migration and proliferation of VSMCs is considered as the key events in both AS and graft vascular diseases. HCMV may be one of important pathogenic factors influencing migration and proliferation of VSMCs.

Homebox (a subset of homeotic genes) are a group of related genes that control the body plan of an embryo along the cranio-caudal (head-tail) axis. It is a highly conservative evolutionary clusters and all their coding products contain a DNA-binding domain composed of about 60 amino acids, which target DNA sequence and modulate gene expression. The HOX gene family exists in all eukaryotes and participates in a variety of activities, such as cell proliferation, apoptosis and cell cycle regulation, etc. Reports showed that HOX gene plays a very important role not only in embryonic development, but in vascular repair, angiogenesis, and tumor metastasis after birth (Gorski and Walsh, 2000; Carè et al., 2001).

Research has shown that HOX genes participate in a variety of cell proliferation and migration by direct or indirect way, i.e., over expression of growth arresting-specific homeobox (Gax) inhibits the proliferation and migration VSMCs induced by serum (Zheng et al., 2014). And the expression of HOXC11 can induce cell proliferation in renal cell carcinoma (Liu et al., 2015). HOXA7 can also stimulate breast cancer cell proliferation by increasing the estrogen receptor alpha (Zhang et al., 2013). In addition, HCMV infection or early antigen of HCMV can induce a variety of abnormal expression of HOX genes (Kadota et al., 1992). According to the information mentioned above, the present study was designed to know whether HOX genes play a role in the occurrence of vascular diseases induced by HCMV.

## Materials and methods

### Materials

Human VSMCs were supplied by Modern Analysis and Testing Center of Central South University. Human embryonic lung fibroblasts (HLF), human HCMV strain AD169, DH-5α, pEGFP-C1 were preserved by Department of Microbiology, Central South University. DMEM and DMEM/F12 (1:1) medium were purchased from Hyclone, USA. Fetal bovine serum (FBS) was from GIBCO. LB broth was from Tianhe Co., Ltd, Hangzhou, China. Ampicillin and kanamycin were from Amresco, USA. Plasmid extraction kit and DNA gel extraction kit were supplied by OMEGA, USA. pGM-T vector kit and PCR master mix were from Tiangen (Beijing) Co., Ltd. TRIzol and Lipofectamine LTX and PLUS reagents were from Life Technologies, USA. cDNA first strand synthesis kit was from Toyobo (Shanghai) biological Technology Co. CCK 8 cell proliferation test kit was supplied by Dongren chemical technology (Shanghai) Co., LTD. Hoechst33342/PI double dye cell apoptosis detection kit was from Beibo (Shanghai) biological technology Co., LTD.

### HCMV preparation

HLF were maintained in DMEM supplemented with 10% FBS, 100 U/mL penicillin and 100 μg/mL streptomycin. Ninety percent confluent HLF cells were infected with 100 μL HCMV for 1 h at 37°C, and then were added DMEM containing 3% FBS and observed daily for cytopathic effects. The cells were subjected to three successive freeze-thaw cycles after obvious (++++) cytopathic effects. Supernatant was harvested followed by centrifugation of 1000 × g to remove cell debris and was done dilutions of 10-time series to do plaques experiment to determine the amount of infectious virus particles. Then the virus was liquated and stored at −80°C until use.

### cDNA profiling

Examination of cDNA profiling was assisted by Shanghai Kang Cheng biological technology limited company. Ninety percent confluent VSMCs were infected with HCMV (MOI = 1). Then the cells were cultured in fresh medium with 3% FBS and harvested at 24, 48, and 72 h post-infection. Briefly, total RNA was harvested using TRIzol reagent (Invitrogen) and the RNeasy kit (Qiagen) according to manufacturer's instructions, including a DNase digestion step. Then the RNAs were amplified and labeled using the Agilent Quick Amp labeling kit and hybridized with Agilent whole genome oligo microarray which contain 41,000+ unique human genes and transcripts in Agilent's SureHyb Hybridization Chambers. After hybridization and washing, the processed slides were scanned with the Agilent DNA microarray scanner using settings recommended by Agilent Technologies. Raw data were normalized using the Quantile algorithm, Gene Spring Software 11.0 (Agilent). Welch's *t*-tests and the Significance Analysis of Microarray tests were used to identify genes that were differentially expressed in the trial subjects of each category, and two standards of statistical value (*P* < 0.01) and fold-change (≥2 or ≤ −2) were used as the filtering criteria. Four samples from the HCMV infection and control groups were analyzed by two-way clustering.

### Real-time PCR

Total RNA was extracted from the above VSMCs using TRIzol (invitrogen, USA) and first-strand cDNA was synthesized using total RNA as a template according to the manufacturer's instructions of the Prime Script RT-PCR Kit (TaKaRa, Dalian, China). One microliter of the reverse-transcripts was added to a 20 μl PCR mixture for 40 cycles with ABI-7500 detection system (Applied biosystems, USA). The primers were as follows: 5′-GGCCAGGTGAGATTCTCCAA-3′ and 5′-TCCATTTAGCGCGTCGATT-3′ (HHEX). The reaction conditions were as follows: a mixture of 2 μL of 10 × PCR buffer, 2 μL of magnesium ion (25 mM), 0.3 μL of dNTPs (25 mmol/L), 0.5 μl of upstream (10 μmol/L) primers, 0.5 μl of downstream (10 μmol/L) primers, Sybr (20 ×) 1 μL, Taq (5 U/μ1) 0.2 μL and cDNA transcripts 1.0 μL. The mixture was incubated at 95°C for 2 min and then 40 cycles of 95°C 10 s and 60°C, 30 s. A melting curve of the reaction system was drawn immediately after the reaction to analyze the specificity of the PCR products. Quantitative analysis of target gene expression data was based on 2^−ΔΔCt^ method. ΔΔCt = (average Ct of target gene-average Ct of β-actin in experimental groups) − (average Ct of target gene − average Ct of β-actin in control group). Four replications of each experimental group, and each sample was tested for three times.

### Construction of HHEX over-expressed vector and transfection

Total RNA was extracted from VSMCs with the Total Extraction Kit (Takara, Dalian, China). First-strand cDNA was synthesized using total RNA as a template according to the manufacturer's instructions of the Prime Script RT-PCR Kit (TaKaRa, Dalian, China). Primers were designed according to the region of the *HHEX* coding region (5′-TAT**GAATTC**GCCACCATGCAGTACCCGCACCC-3′, *EcoR I* site underlined; and 5′-CGCGCG**GGATCC**TCCAGCATTAAAATAGCT-3′, *Bam HI* site underlined). PCR amplification was performed using 1 μl first-strand cDNA as a template in 20 μl PCR mixture. The PCR conditions were an initial 94°C for 2 min; 40 cycles of 94.0°C for 0.5 min, 56°C for 0.5 min and 72°C for 1 min; and an extension at 72°C for 10 min. The PCR products were purified using a DNA gel extraction kit (Sangon, Shanghai, China), then ligated to pGM-T vector and transformed into DH-5α for sequencing. The Fragments encoding the HHEX were digested from pGM-T with *EcoR1* and *BamH1* and cloned into *EcoR I* and *BamH I* sites of plasmid pEGFP-C1. The constructed plasmid was transformed into DH-5α and verified by restriction enzyme mapping and DNA sequencing (Huada, Shanghai, China).

VSMCs were cultured in 6-well plate in DMEM/F12 (1:1) supplemented with 12% FBS at 37°C under 5% CO_2_ in humidified air. 90% confluent monolayer cultures were cultured in DMEM/F12 without serum at 37°C under 5% CO_2_ for 24 h, then washed twice by invitrogen optiMEM medium, and added 1.5 ml optiMEM in every well. 5 μg plasmid was added to 250 μl optiMEM at room temperature for 10 min after mixing, then 5 μl plus reagent was added and mixed. In another tube, 15 μl LTX reagent was added to 250 μl optiMEM medium at room temperature for 30 min after mixing. Then the two tubes were mixed and the complex was added to cells drop by drop, gently mixed, then incubated at 37°C and 5% CO_2_ for 6 h. Fresh media was added 6 h later.

### Flow cytometry

Cells were harvested at 24, 48, and 72 h after transfection and fixed in 75% ethanol. The fixed cells were washed twice with phosphate-buffered saline, stained in a propidium iodide solution (50 μg/ml) for 1 h, and treated with a ribonuclease A solution (20 ug/ml) for 30 min for proliferation assay. Cells were labeled with 150 μl of hoechst33342 and 75 μl of PI for 10 min in darkness and at room temperature for apoptosis assay. Normal living cells and early apoptotic cells could resist the staining by PI, but necrotic cells could not. Flow cytometry was carried out by tumor research institute, Central South University.

### Proliferation assay

The recombinant plasmid pEGFP C1/HHEX was transfected into human VSMCs, and digested to replant into 96-well plate with 10^3^ cells/well, and incubated at 37°C 5% CO_2_ for 4, 8, 12, 24, 48, 72 h, respectively. 10 μl of CCK 8 reagent was added to obtained cells at different time points and incubated for 3 h, then OD 450 nm was recorded and the cell growth curve was plotted. Four independent experiments were performed.

### RT-PCR assay

Total RNA was extracted from VSMCs with the Total Extraction Kit (Takara, Dalian, China). First-strand cDNA was synthesized using Prime Script RT-PCR Kit (TaKaRa, Dalian, China). The amplification primers, conditions and product length used for caspase2, caspase3, CDK2, CDK6, cyclin B2, and cyclin D2 were listed in Table 1.

**Table 1:** **Primers used in the experiments**.

**Genes**	**Primers (5′–3′)**	**Reaction conditions**	**Cycles**	**Products (bp)**
Caspase2	Upstream: TGCCCAAGCCTACAGAAC Downstream: TGTGCCAGGAGCATAACC	94°C 40 s	32	360
		52°C 40 s		
		72°C 40 s		
Caspase3	Upstream: GGAATTGATGCGTGATGT Downstream: ACCAGGTGCTGTGGAGTA	94°C 40 s	32	316
		52°C 40 s		
		72°C 40 s		
CDK2	Upstream: CCGCCTGGACACTGAGACT Downstream: GTGGAGGACCCGATGAGA	94°C 40 s	30	271
		56°C 40 s		
		72°C 40 s		
CDK6	Upstream: TAACCTCAGTGGTCGTCAC Downstream: GTCTTTGCCTAGTTCATCG	94°C 40 s	30	299
		50°C 40 s		
		72°C 40 s		
Cyclin B2	Upstream: ATGTGACTATTAGGCGAACT Downstream: AGAGCAAGGCATCAGAAA	94°C 40 s	30	264
		47°C 40 s		
		72°C 40 s		
Cyclin D2	Upstream: ATTTACACCGACAACTCCATC Downstream: CTCAGTCAGGGCATCACAA	94°C 40 s	30	318
		55°C 40 s		
		72°C 40 s		

The reaction conditions (50 μl) were as follows: a mixture of 25 μL of 2 × PCR master mix, 1 μl of upstream (10 pmol/L) primers and downstream (10 pmol/L) primers respectively, cDNA transcripts 3.0 μL. The mixture was incubated at 94°C for 2 min and then 40 cycles, and 72°C for 10 min. GAPDH was used as control (GAPDH:5′-ACCACAGTCCATGCCATCAC-3′, 5′-TCCACCACCCTGTTGCTGTA-3′, 450 bp). Scan gray scale of the bands and calculate relative gene expression level using NIH image J software. Relative gene expression level = gray value of tested genes/gray value of GAPDH. Six independent experiments were performed.

### Statistical analysis

Data are expressed as means ± standard errors of the means. Analysis was performed using SPSS 12.0 for windows. Statistical significance was tested using either *t*-test between two samples or single factor analysis of variance among multiple samples. A *P* < 0.05 was considered statistically significant.

## Results

### The infection of HCMV to VSMCs

**Figure 1:**
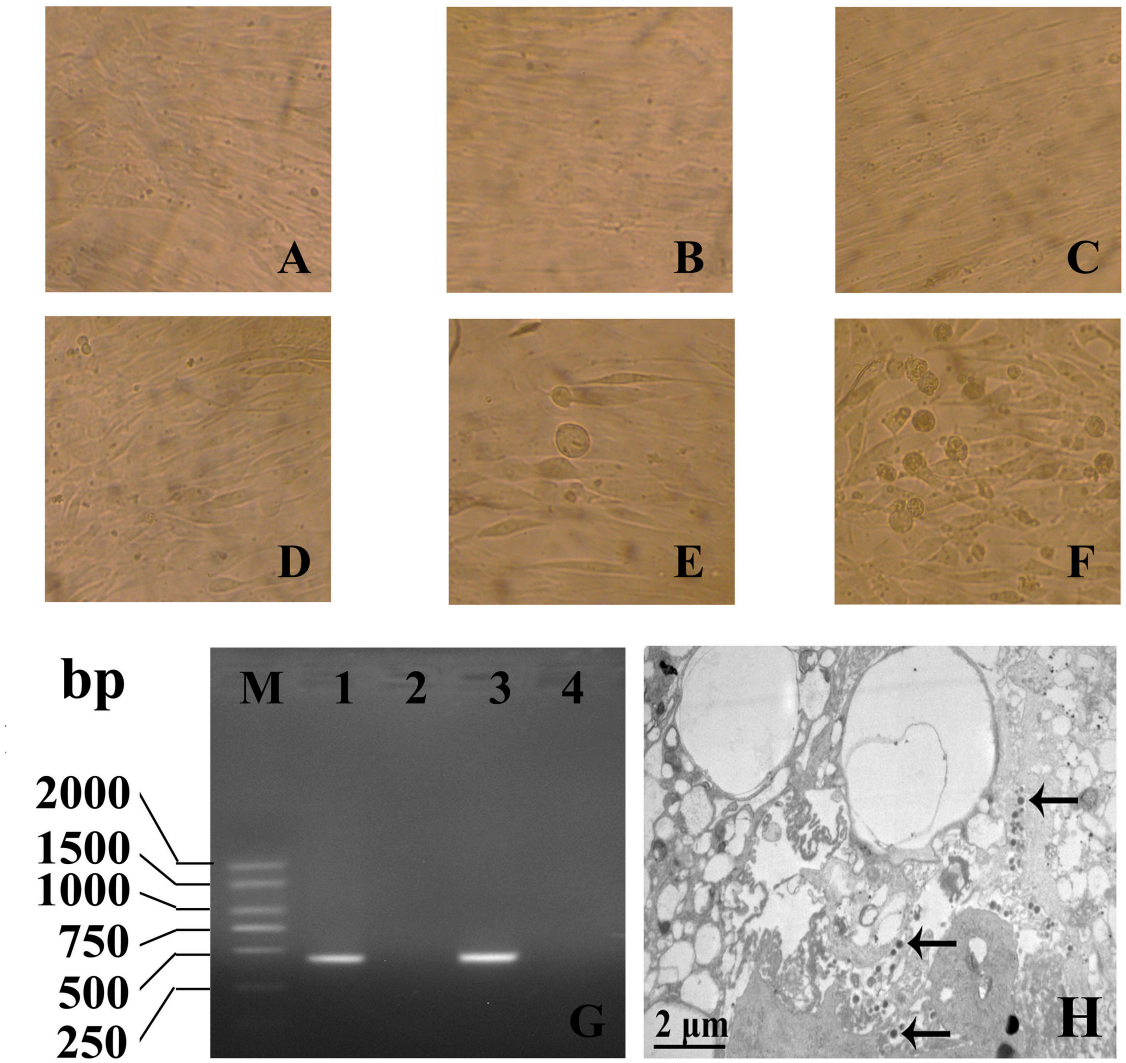
**The infection of HCMV to VSMCs was assayed by direct inverted microscope (A–F)**, RT-PCR **(G)** and electron microscopic observation **(H)**. **(A–C)** 1, 4, 8 days of mock infection group; **(D–F)** 1, 4, 8 days of HCMV infection group (× 400); **(B)** HCMV IE gene could be amplificated from VSMCs infected with HCMV. M: DL2000 plus DNA marker; 1: IE gene PCR products; 2: Mock infection 3: Positive control; 4: Negative control; **(C)** On the third day after infection, the viral particles were visible within the cell (Arrow, × 10,000).

The human VSMCs were cultured in DMEM/F12 and inoculated with HCMV. RT-PCR for detecting HCMV IE gene and electron microscopy for detecting intact viral particles were used to study the infection of human VSMCs by HCMV. The results showed that 24 h after infection, some individual cells appeared swelling. With prolonged incubation time, cytopathic effects become more and more serious and eighth days after infection, almost all the cells showed cytopathic effects (Figures 1A–F). Using the technology of RT-PCR, HCMV IE gene could be amplificated from VSMCs infected with HCMV (Figure 1G). The real PCR products were confirmed by direct sequencing. On the third day after infection, the cells were harvested and examined by electron microscopy. The results showed the viral particles were visible within the cell (Figure 1H). These results showed that HCMV infects human VSMCs, and replicates intact virus particles in the cells.

### HOX gene expression in VSMCs after HCMV infection

**Figure 2:**
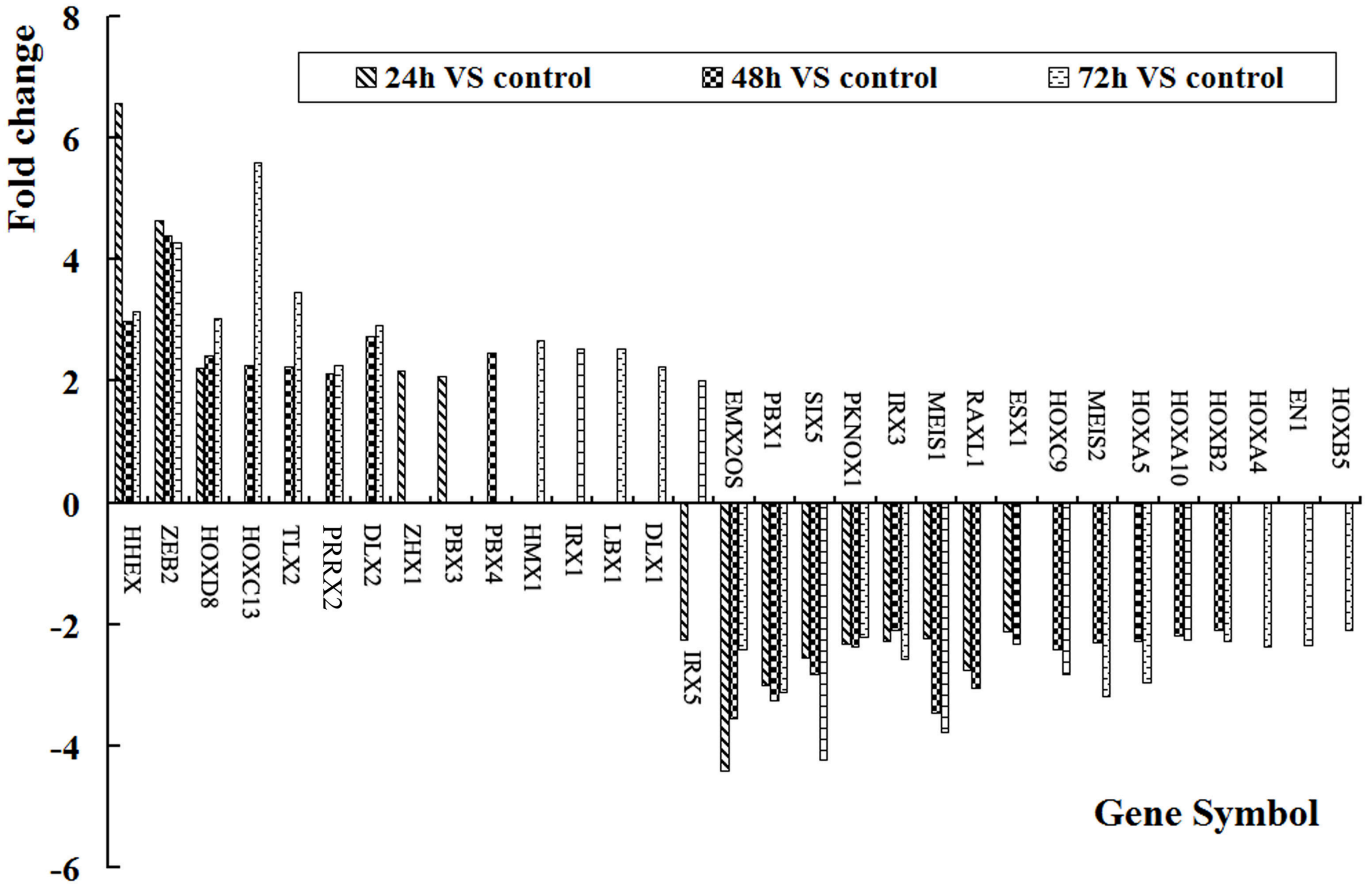
**HOX gene expression levels in VSMCs after HCMV infection**. There were 31 significant differences of HOX genes.

Expression levels of HOX genes were detected by cDNA profiling technology after 24, 48, and 72 h of HCMV infection. Compared with mock control, more than 2-fold of change were recognized as deferentially expressed genes. The results showed that at 24 h after infection, there were 15 differential expression of HOX genes with 5 up-regulated and 10 down-regulated; at 48 h after infection, 22 HOX genes deferentially expressed, including 9 up-regulated and 13 down-regulated; at 48 h after infection, 27 HOX genes differentially expressed, including 13 up-regulated and 14 down-regulated. There were 9 differentially expressed HOX genes: HHEX, ZEB2, HOXD8, EMX2OS, SIX5, PKNOX1, IRX3, MEIS1, and ESX1 in all three tested groups with 3 up-regulated and 6 down-regulated genes. HHEX increased at 24, 48, and 72 h after HCMV infection with more than three-fold, as shown in Figure 2.

### HCMV infection promoted HHEX expression

**Figure 3:**
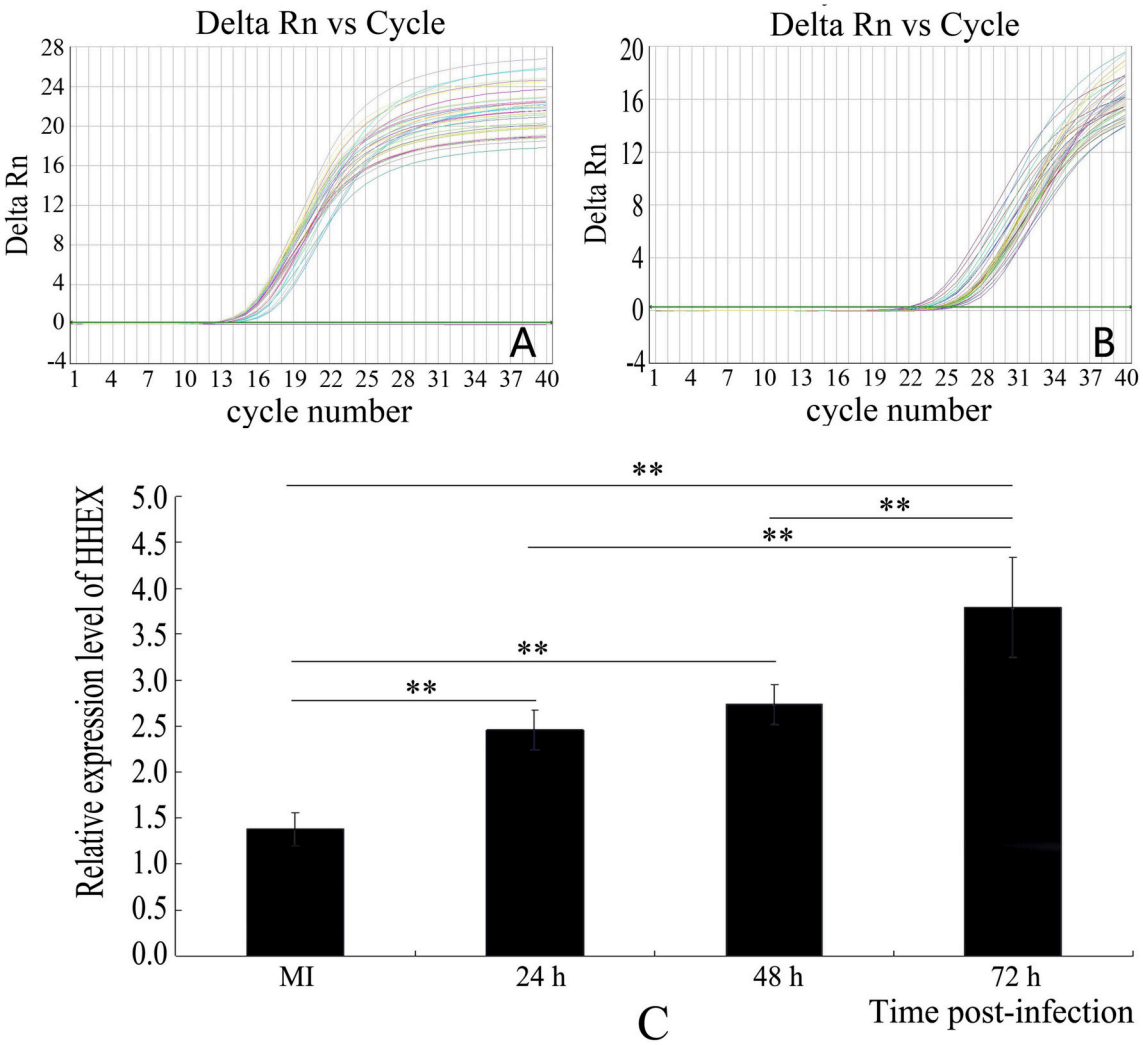
**Expression of HHEX was assayed by real-time PCR. (A)** β-actin amplification curve; **(B)** HHEX amplification curve; **(C)** Relative mRNA expression of HHEX. The results showed that HCMV promotes the expression of HHEX (*n* = 4, ^**^P < 0.01).

Real time PCR was used to validate the HHEX expression after HCMV infection. The results showed that HHEX gene expression were significantly higher at 24, 48, and 72 h after infection (*F* = 36.36, *p* < 0.01) when compared with mock-infection group (Figure 3), indicating that HCMV infection promotes HHEX gene expression.

### HHEX promotes proliferation of VSMCs

**Figure 4:**
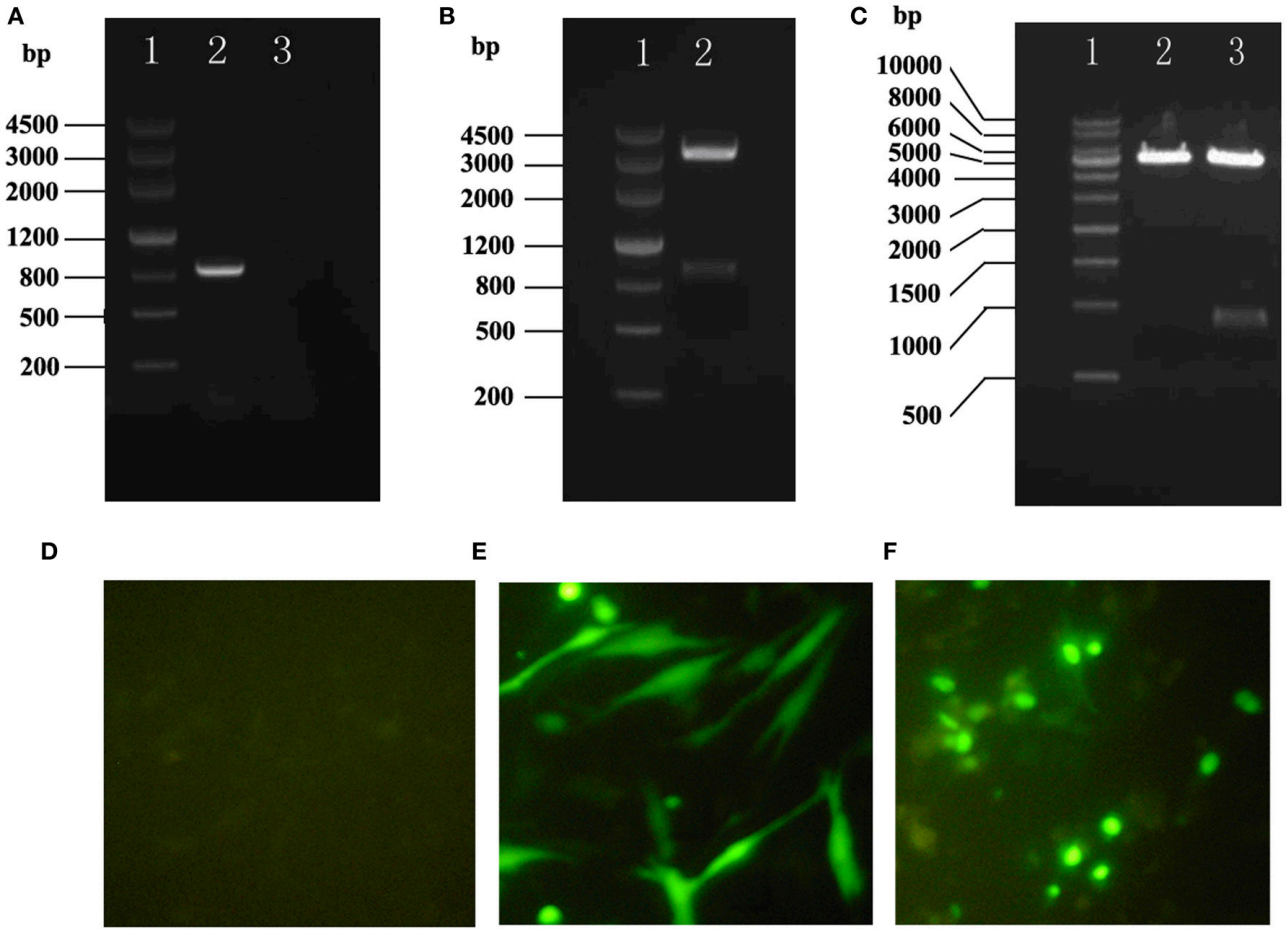
**Construction and assay of pEGFP-C1/HHEX. (A)** HHEX cDNA electrophoresis products of PCR (1: DNA marker III, 2: HHEX cDNA, 3: negative control); **(B)** pGM-T/HHEX digestion products (1: DNA marker III, 2: pGM-T/HHEXdigestion products); **(C)** pEGFP-C1/HHEX digestion products (1: 1Kb DNA marker; 2: pEGFP-C1 digestion products; 3: pEGFP-C1/HHEX digestion products); **(D)** Normal control; **(E)** pEGFP-C1; **(F)** pEGFP-C1/HHEX.

pEGFP-C1/HHEX was constructed (Figures 4A–C) and transfected into VSMCs. After 24 h transfection, the successful transfection was observed by fluorescence microscopy. The results showed that no fluorescence was observed in control group while visible fluorescence in cytoplasm was observed in empty plasmid group, and obvious fluorescence was observed in pEGFP C1/HHEX group, which located in the nucleus (Figures 4D–F).

**Figure 5:**
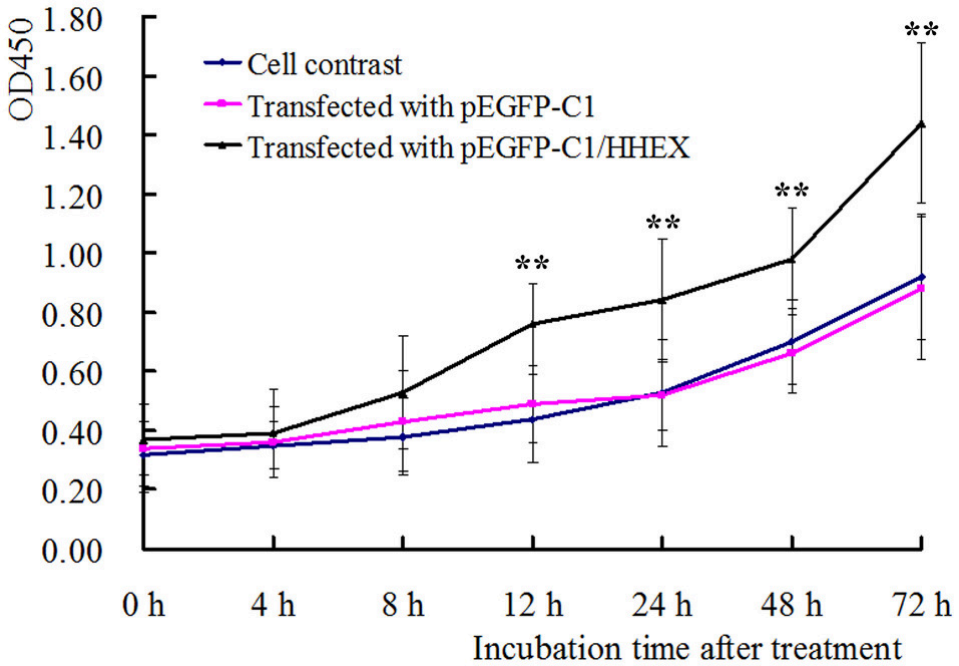
Proliferation of VSMCs was assayed by CCK-8 kit (*n* = 5, ^**^P < 0.01). HHEX over expression can promote proliferation of VSMCs.

The recombinant plasmid pEGFP C1/HHEX was transfected into human VSMCs, and digested to replant into 96-well plate for proliferation assay by using CCK8. The results showed that the cells in the control group and the empty plasmid group had similar cell proliferation curve, but the cells in HHEX over-expressed group, were significantly higher than the cells in the control group and the empty plasmid group from 12 to 72 h after transfection (Figure 5), indicating that HHEX can obviously promote VSMCs proliferation.

### HHEX inhibits apoptosis and promote proliferation of VSMCs

**Figure 6:**
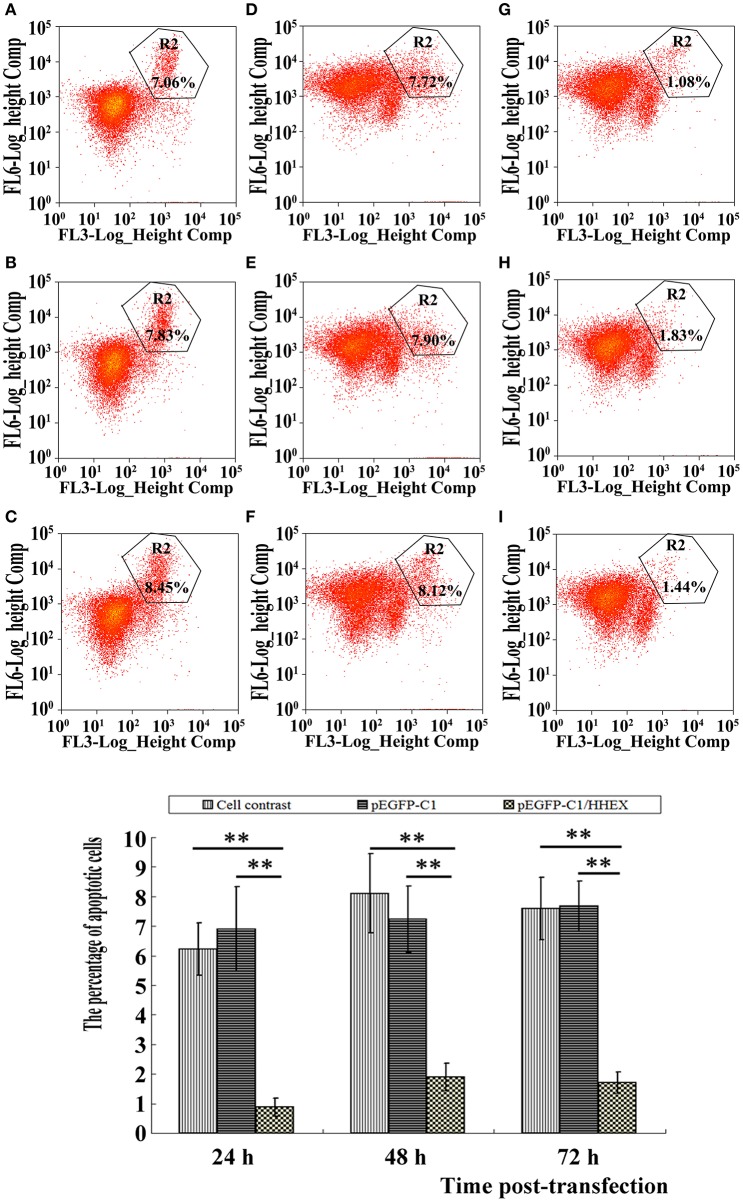
Apoptosis was assayed by flow cytometry (*n* = 4, ^**^P < 0.01). (A–C) Normal control; **(D–F)** pEGFP-C1 transfection; **(G–I)** pEGFP-C1/HHEX transfection; **(A,D,G)** 24 h post-infection; **(B,E,H)** 48 h post-infection; **(C,F,I)** 72 h post-infection. HHEX over expression can inhibit VSMCs apoptosis.

The apoptosis of VSMCs were detected by using flow cytometry technique and apoptosis rates in the control group and the empty plasmid group at 24 h were 6.23 ± 0.89 and 6.90 ± 1.44; at 48 h were 8.11 ± 1.12 and 7.23 ± 1.34; at 72 h were 7.59 ± 1.05 and 7.67 ± 0.86. There was no significant difference (*F* = 2.67 and 0.55, *p* > 0.05), but apoptosis rates in HHEX over-expressed group at 24, 48, and 72 h were 0.88 ± 0.30, 1.90 ± 0.47, and 1.71 ± 0.35, which were significantly lower than in the control group and the empty plasmid group (Figure 6), indicating that HHEX expression can inhibit apoptosis of VSMCs.

**Figure 7:**
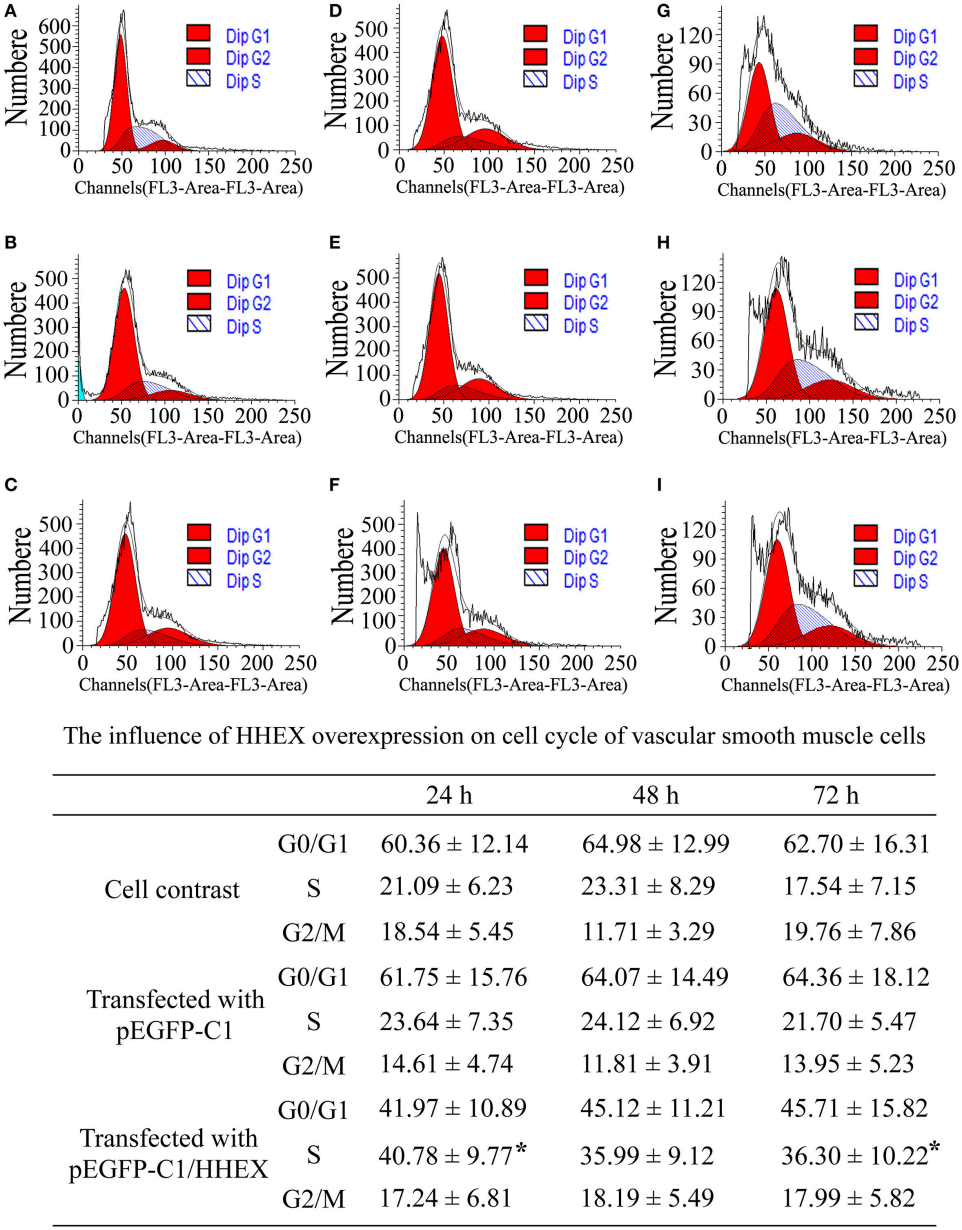
Influence of HHEX on cell cycle of VSMCs was assayed by flow cytometry (*n* = 4, ^*^P < 0.05). (A–C) Normal control; **(D–F)** pEGFP-C1) transfection; **(G–I)** pEGFP-C1/HHEX transfection; **(A,D,G)** 24 h post-infection; **(B,E,H)** 48 h post-infection; **(C,F,I)** 72 h post-infection. HHEX over expression can promote G0/G1 phase into S phase.

The results of cell cycle showed that G2/M phase had no differences among various treatments at 24, 48, and 72 h (*F* = 1.18, *p* = 1.18). G0/G1 phase also had no differences among various treatments at 24, 48, and 72 h (*F* = 1.76, *p* = 1.76). However, ratio of S phase cells in the HHEX over-expressed group was significantly higher than in the control group and the empty plasmid group at 24 h (*F* = 7.31, *p* = 0.013) and 72 h (*F* = 6.28, *p* = 6.28), indicating that the HHEX over expression can promote G0 / G1 phase cells into S phase and VSMCs proliferation (Figure 7).

### Expression of apoptosis and proliferation-associated genes

**Figure 8:**
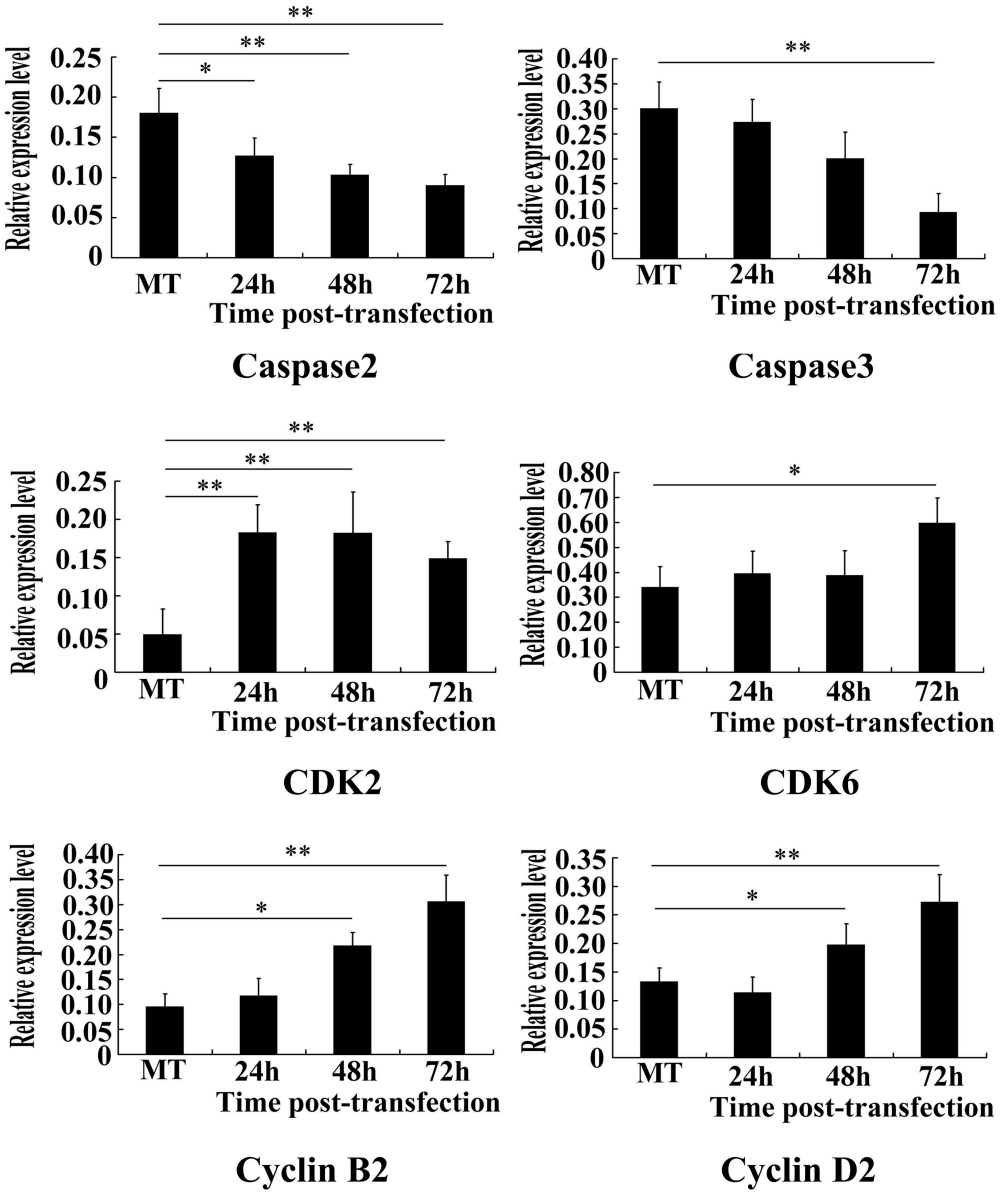
Apoptosis and proliferation-associated genes were assayed by RT-PCR (*n* = 6, ^*^P < 0.05; ^**^P < 0.01). Results showed that caspase2 and caspase3 expression obviously decreased, while CDK2, CDK6, Cyclin B2, and Cyclin D2 increased).

The results of RT-PCR showed that when compared with pEGFP-C1, Caspase 2 expression in pGEFP- C1/HHEX transfection group decreased at various time points (*F* = 13.96, *p* = 0.003) and caspase 3 expression decreased at 72 h (*F* = 15.11, *p* = 0.0002) (Figure 8). Relative expression level of CDK2 at 24, 48, and 72 h were significantly higher than those in control group (*F* = 13.39, *p* = 0.0004); CDK6 relative expression level had no differences at 24 and 48 h but increased at 72 h (*F* = 16.02, *p* = 0); the relative expression level of cyclin B2 and cyclin D2 had no differences at 24 h, but increased at 48 and 72 h (*F* = 29.18 and 16.87, *p* < 0.01) (Figure 8).The results showed that over expression of HHEX may inhibit apoptosis of VSMCs by inhibiting the expression of Caspase 2 and Caspase 3 and promote the proliferation of the cells by promoting expression of cyclins and cell cycle dependent kinase.

## Discussion

HCMV is one member of beta herpes virus subfamily with common infection in adults. Studies have shown that global infection rate of HCMV is 60–90% with latency in the body for life long after primary infection and repeated recurrence. The virus is not only closely associated with embryo deformity, also with a variety of cardiovascular diseases, especially blood vessel proliferative diseases including AS, hypertension and graft vascular disease (graft AS) (Cheng et al., 2009; Lee et al., 2014). However, the mechanism of these diseases induced by HCMV has not fully elucidated.

VSMCs is one of the main cellular ingredients involved in blood vessel proliferative diseases. HCMV can infect VSMCs and cause hyperplastic infection (Tumilowicz et al., 1985). Moreover, HCMV can induce cell proliferation and migration through its immediate early gene expression (Lemström et al., 1993; Zhou et al., 1999), which was confirmed by experimental animal models (Strååt et al., 2009), indicating HCMV participates in the two key process of hyperplastic diseases, VSMCs proliferation and migration to the intima. CMV-IE protein promotes the production of interleu-kin-1 and tumor necrosis factor-a, and these cytokines induce PDGF B-chain gene expression in the endothelial cells. At the same time, besides VSMCs, HCMV can infect almost all other cell types associated with blood vessel hyperplastic diseases, such as vascular endothelial cells (Alcendor et al., 2012), fibroblasts (Shanley et al., 1996), macrophages (Strååt et al., 2009) and so on, and promote the proliferative disease occurrence and development through direct or indirect way.

HOX gene, as a transcription factor, can combine with target DNA, regulating downstream gene expression involved in angiogenesis and repair, which closely related to proliferative vascular disease pathogenesis (Zheng et al., 2014). So, whether vessel proliferative disease induced by HCMV is related with HOX genes? The present study showed that HCMV results in significant difference of nine HOX genes: HHEX, ZEB2, HOXD8, EMX2OS, SIX5, PKNOX1, IRX3, MEIS1, and ESX1, especially HHEX gene by increase of more than three-folds at 24, 48, and 72 h after infection. HHEX, namely the orphan HOX gene (also called haematopoietically expressed HOX or proline-rich homeodomain), can be a transcription inhibitor or transcription activator, plays an important role in the process of vertebrate development. In humans, the genes locates in the chromosome 10. Studies have shown that HHEX is necessary at the beginning of an endothelial cell differentiation and the whole process of hematopoietic differentiation, and homozygous mutation of HHEX induced mid pregnancy death because of severe defects in the formation of forebrain, thyroid, liver, B cells, heart, and blood vessels (Martinez Barbera et al., 2000).

Studies have shown that overexpression of HHEX can cause vascular endothelial cell proliferation (Hallaq et al., 2004). Can the excessive expression of HHEX after HCMV-infected VSMCs promote VSMCs proliferation? However, the mechanism is not well understood, the present study suggested that HHEX over expression in VSMCs can promote the cell proliferation and inhibited cell apoptosis. In addition, HHEX inhibited the expression of apoptosis-associated caspase2 and caspase3 and promoted the expression of cell cycle-related genes, such as CDK2 and CDK6, CyclinB2, and CyclinD2, indicating HHEX expression can lead to proliferation and apoptosis imbalances of VSMCs through many kinds of genes. Moreover, numerous studies have demonstrated that HHEX modulate the expression of many kinds of important vascular injury and remodeling-associated genes, including smooth muscle myosin heavy chain (MHC), fibrinolytic enzyme original activators inhibitor 1 (PAI 1), nitric oxide synthase (iNOS), early growth response factor 1 (Egr-1), platelet-derived growth factor A (PDGF), and vascular endothelial growth factor and receptor (VEGF receptor), etc. Therefore, HCMV play an important role in occurrence and development of blood vessel proliferative diseases.

## Author contributions

SL, LC, and GW conceived the experiments; LL, ML, LK, YL, ZD, and BW performed the experiments; YT and GW analyzed the data; YT wrote the manuscript.

### Conflict of interest statement

The authors declare that the research was conducted in the absence of any commercial or financial relationships that could be construed as a potential conflict of interest.
